# 筛选高表达CPEΔN的H1299肺癌细胞株

**DOI:** 10.3779/j.issn.1009-3419.2015.06.03

**Published:** 2015-06-20

**Authors:** 静 孙, 桂荣 张, 虹月 王, 慧 申

**Affiliations:** 110042 沈阳，辽宁省肿瘤医院生物治疗研究中心，辽宁省肿瘤研究所 Biotherapy Research Center, Liaoning Cancer Hospital and Institute, Shenyang 110042, China

**Keywords:** 肺肿瘤, 羧肽酶EN端截短体, 慢病毒载体, Lung neoplsms, N-terminal truncated carboxypeptidase E, Lentiviral vector

## Abstract

**背景与目的:**

N端截短的羧肽酶E（N-terminal truncated carboxypeptidase E, CPEΔN）是一个新的肿瘤转移相关蛋白。本研究旨在筛选高表达CPEΔN的H1299肺癌细胞株，为完成小鼠活体成像实验创造条件。

**方法:**

构建CPEΔN的慢病毒表达载体。分别用CPEΔN慢病毒表达载体或对照慢病毒空载体转染H1299细胞，2 μg/mL的嘌呤霉素加压筛选。Western blot分析CPEΔN蛋白的表达，荧光素酶报告基因实验分析荧光素酶对底物的分解作用。

**结果:**

当感染倍数（multiple of infection, MOI）是20时，慢病毒对H1299细胞的转染效率可以达到80%。CPEΔN高表达H1299细胞株（H1299-CPEΔN）和对照慢病毒载体表达H1299细胞株（H1299-control）中CPEΔN蛋白的表达量为4:1。H1299-CPEΔN和H1299-control均能够有效分解荧光素酶底物，可以满足活体成像实验的需求。

**结论:**

筛选出高表达CPEΔN的H1299肺癌细胞株，为活体成像实验的开展创造了条件，也为进一步解释CPEΔN促进肿瘤转移的分子机制奠定了基础。

## Abstract (en)

**Background and objective:**

Te N-terminal truncated carboxypeptidase E (CPEΔN) protein is a novel biomarker of tumor metastasis. Tis study screened the H1299 cell line with a highly expressed CPEΔN gene for *in vivo* imaging experiment.

**Methods:**

Human CPEΔN gene was cloned into the luciferase lentiviral vector. H1299 cells transduced with CPEΔN or control lentiviral vectors were selected with 2 μg/mL puromycin. Te expression of CPEΔN was identifed through Western blot analysis, and luciferase activity was measured using luciferase reporters.

**Results:**

Te human CPEΔN lentiviral expression vector was successfully constructed. Te transfection rate of H1299 cells by the lentivirus achieved 80%, with an infection multiplicity of 20. Te H1299 cell line with high CPEΔN (H1299-CPEΔN) expression was established, with an increase in CPEΔN expression by four times compared with the control lentivirus-transfected H1299 cell line (H1299-control). As H1299-CPEΔN and H1299-control can e?ectively decompose luciferase substrates, they can be applied in in vivo imaging.

**Conclusion:**

H1299-CPEΔN and H1299-control can be used in *in vivo* imaging experiment for further research on molecular mechanisms and signal transduction to elucidate the role of CPEΔN in lung cancer metastasis.

羧肽酶E（carboxypeptidase E, CPE）是一个金属离子依赖的肽链端解酶，它的生物学功能非常多元化。按照亚细胞定位的差异，CPE的功能可以被分为外肽酶功能和非酶功能两类^[1]^。第一部分CPE是以分泌形式表达的，这部分CPE主要分布在内分泌及神经内分泌细胞中，他们可以将激素原或神经多肽裂解为成熟的激素或神经肽，进而调节内分泌及神经信号的传导^[2-8]^。第二部分CPE是以非分泌性形式存在的，他们或者结合在细胞质膜上参与高尔基体的转运过程^[9]^；或者定位在细胞质、细胞核中参与细胞信号传导、转录调控。这部分CPE蛋白的功能是多元的，也是目前最受关注的。

近年研究者采用信使RNA（messenger RNA, mRNA）芯片技术分析了CPE在不同肿瘤细胞中的表达，结果显示在神经内分泌起源的肿瘤组织如成神经细胞瘤、胶质瘤中CPE的高表达预示肿瘤具有较好的预后^[10-12]^，而在一些非内分泌的肿瘤如乳腺癌、肾细胞癌、宫颈癌中^[13-17]^，CPE的高表达常常预示着患者的生存期短、预后差。在不同起源的肿瘤组织中CPE的功能是矛盾的且不明朗。2011年香港大学Lee的一项研究给我们带来了新的启示，他们在肝细胞癌中发现一种截短形式的CPE蛋白即CPE△N，他可以作为一个独立性指标，预测肝癌和嗜铬细胞瘤的转移和复发^[18, 19]^。CPE△N能够与组蛋白去乙酰化酶1和组蛋白去乙酰化酶2结合进而抑制转移相关蛋白NEDD9的表达。

2013年周坤等^[20, 21]^在结直肠癌中也发现了CPE△N的表达，并且证实在肿瘤组织中只有截短形式的CPE△N表达，CPE△N高表达的病例复发、转移率高于CPE△N低表达病例。肝细胞癌、嗜硌细胞瘤、结肠癌是起源不同的三种肿瘤类型，CPE△N高表达可以预测三者的复发，提示CPE△N很可能是一个广谱的肿瘤预测分子。

在本研究室的前期工作中，我们在9个肺癌细胞株中检测到CPE△N的表达，并且证实CPE△N的表达可以加速肺癌细胞浸润转移。为进一步明确CPE△N促进肺癌转移的易发部位，并揭示其可能分子机制，本研究选取CPE△N蛋白表达量最少的肺癌细胞株H1299作为母细胞，筛选得到了共表达荧光素酶和CPEΔN的H1299肺癌细胞株，并证实CPEΔN高表达细胞株和对照细胞株均可以有效分解荧光素酶底物，可以用于后期的小鼠活体成像实验，现将结果报道如下。

## 1 材料和方法

### 1.1 材料

慢病毒载体pCMV-GFP-LV、pLenti-CMV-MCS-HA-3Flag-P2A-LUC-T2A-Puro和慢病毒包装试剂盒购自上海禾元生物；高保真酶、胶回收试剂盒、质粒抽提试剂盒购自天根生物；限制性内切酶、T4连接酶来自美国NEB公司；SYBR Premix EX Taq试剂盒购自TaKaRa，感受态细胞DH5α购自TaKaRa；H1299细胞购自上海细胞库；DMEM培养基、胎牛血清和青、链霉素购自天根生物；转染试剂脂质体2000购自Invitrogen，嘌呤霉素（Puromycin）购自翊圣公司，双荧光报告基因检测试剂盒购自Promega。

### 1.2 方法

#### 1.2.1 确定慢病毒载体对H1299细胞的转染效率

将H1299细胞接种到24孔板中，细胞数为1×10^4^/孔。约16 h后，用带荧光标记的慢病毒载体pCMV-GFP-LV转染H1299。病毒量按如下公式计算：细胞数×感染倍数（multiple of infection, MOI）值/病毒原液滴度×10^3^=病毒加药量。本研究的MOI值共设计了5个梯度，分别是MOI 10、MOI 20、MOI 40、MOI 80和MOI 100。完成转染后加入puromycin，使其终浓度为5 μg/mL，20 h后更换为新鲜培养基。72 h后荧光显微镜拍照，确定最佳感染条件。

#### 1.2.2 构建CPEΔN慢病毒表达载体

CPEΔN正向引物为CGAGCTCAAGCTTCGAATTCGCCACCATGAGGCG GCGCCGGCG（含同源重组序列、kozak序列、*Eco*R I酶切位点和目的基因5’端配对序列）；反向引物为：TCATCCTTGTAGTCGGATCCAAAATTTAAAGT TTCTGACATCAT（含同源重组序列、*Bam*H I酶切位点，目的基因3’端配对序列）。扩增CPEΔN基因后，用*Eco*R I和*Bam*H I酶切处理，之后与相同酶切处理的pLenti-CMV-MCS-HA-3Flag-P2A-LUC-T2A-Puro载体进行同源重组，转化大肠杆菌DH5α感受态细胞。菌落PCR筛选阳性克隆，之后酶切鉴定并验证表达。

#### 1.2.3 筛选H1299-CPEΔN和H1299-control细胞株

将H1299细胞接种到24孔板中，细胞数为1×10^4^/孔，细胞汇合度约30%；16 h后分别转染CPEΔN慢病毒表达载体或慢病毒空载体，MOI值取20，Puromycin终浓度为5 μg/mL，24 h后换液，弃去培养基每孔加入500 μL的新鲜培养基。72 h以后，加入含2 μg/mL puromycin的新鲜培养基。每隔2-3天换液1次，药物筛选约14 d。获得高表达CPEΔN和对照慢病毒载体的H1299肺癌细胞系（分别命名为H1299-CPEΔN和H1299-control）。通过Real-time PCR和Western blot分析CPEΔN的表达，并冻存H1299-CPEΔN和H1299-control细胞株。

#### 1.2.4 荧光素酶报告基因检测

按照荧光素酶报告基因检测试剂盒说明，裂解H1299-CPEΔN和H1299-control细胞株，检测二者分解荧光素酶底物的活性。

## 2 结果

### 2.1 慢病毒载体可以高效感染H1299肺癌细胞

为了分析慢病毒载体对H1299细胞的感染效率、优化感染条件，本研究选择了一个带绿色荧光蛋白，并且转染效率和pLenti-CMV-MCS-HA-3Flag-P2A-LUC-T2A-Puro相同的慢病毒载体pCMV-GFP-LV作为研究对象。设计了MOI 10、MOI 20、MOI 40、MOI 80、MOI 100共5个病毒转染梯度，结果显示当MOI 20、MOI 40、MOI 60、MOI 80、MOI 100时感染效率都达到80%以上（图 1）。本着高效、低毒的原则，选择MOI 20作为后续病毒感染的最佳条件。

**1:**
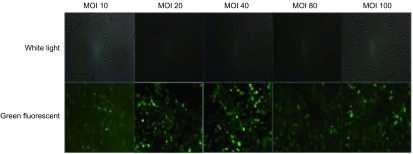
分析不同MOI条件下，慢病毒载体对H1299细胞的转染效率 The transfection efficiency of lentiviral vector in H1299 cells at different multiple of infection (MOI)

### 2.2 分析CPEΔN蛋白的表达并确定其对荧光素酶底物的分解活性

通过菌落PCR和酶切鉴定，证实pLenti-CMV-MCS-HA-3Flag-P2A-LUC-T2A-Puro-CPEΔN慢病毒表达载体构建成功（图 2A）。加入嘌呤霉素加压筛选14 d后，获得了H1299-CPEΔN和H1299-control细胞株。通过Western blot和Real-time PCR检测两个细胞株中CPEΔN的表达。结果如图 2B所示，二个细胞系中CPEΔN蛋白表达量约为4:1（凝胶灰度扫描结果），CPEΔN mRNA的比值约为2:1。

**2:**
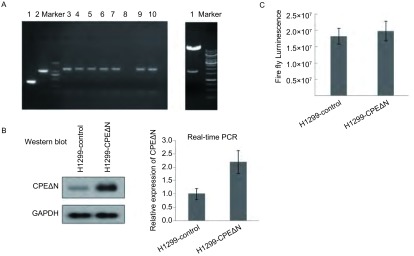
CPEΔN表达载体的构建及鉴定。A：左图：菌落PCR鉴定重组CPEΔN表达载体。阴性对照（第1道），阳性对照（第2道），挑取8个菌落克隆进行菌落PCR鉴定（3道-10道）；右图：酶切鉴定重组CPEΔN表达载体；B：Western blot和Real-time PCR分析CPEΔN蛋白在CPEΔN高过表达细胞系和对照H1299细胞系中的表达；C：报告基因检测分析H1299-CPEΔN和H1299-control细胞株中荧光素酶活性。 Construction and identification of human CPEΔN expression vector. A: Left picture, Identification of recombinant CPEΔN expression vectors using colony PCR. Negative control (lane 1), positive control (lane 2), 8 colonies picked for colony-PCR identification (lanes 3-10). Right picture, identification of recombinant CPEΔN expression vectors by restriction enzyme; B: Analysis of CPEΔN expression by Western blot and real-time PCR in H1299-CPEΔN cells and H1299-control cells; C: Luciferase reporter gene assay in H1299-CPEΔN cells and H1299-control cells.

分析两个细胞系对荧光素酶底物的分解作用，每组取1×10^4^个细胞，CPEΔN高表达细胞株的luciferase均值为19, 891, 000，对照慢病毒载体细胞株的luciferase均值为18, 281, 000（图 2C），二者分解荧光素酶底物的能力没有显著差异。提示筛选到的H1299-CPEΔN细胞株在表达CPEΔN的同时，能够有效地分解荧光素酶底物可以用于后续的活体成像实验。

## 3 讨论

CPEΔN是2011年被鉴定出来的肿瘤转移相关蛋白。Lee等^[18]^证实在所有肝癌病例只表达CPE△N。当癌和癌旁正常组织CPE△N RNA比值> 2，术后2年复发率高达92.8%，中位生存时间为8.6个月；而癌和癌旁CPE△N RNA比值< 2的病例，术后2年复发率只有25.2%，中位生存时间超过90个月，组间差异具有统计学意义。2013年Zhou等^[20]^再次证实CPEΔN的高表达可以作为独立性指标预测结直肠癌的预后不良。在这些文献的提示下，本课题组分析了CPEΔN表达与肺腺癌预后的相关性也得到相似结论结果将在另外文章发表。

为了探索CPEΔN过表达，是否像我们预测的那样可以加速肿瘤细胞向脑、肝脏或骨组织这些代谢、增殖旺盛的区域转移，本课题设计了小鼠活体成像实验。为了尽可能地追踪肿瘤细胞去向，去除动物本底干扰，我们选择了一个经过改造的慢病毒表达载体。选择用这个表达载体主要基于3个考虑：①慢病毒载体的转染效率可以达到80%-90%，并且这种基因整合可以随着细胞分裂被传递到子代；②这个载体将目的基因和荧光素酶基因偶联在同一个启动子上，可以实现一个细胞同时表达两种蛋白，不必同时转染两种质粒再用两种抗生素加压筛选，方法学上简单、易行；③小鼠体内不表达荧光素酶，这样可以最大程度的去除本底。通过腹腔内注入荧光素酶底物，在30 min能够清晰显示肿瘤细胞在小鼠体内的分布情况。据报道通过荧光检测技术可以追踪到腹腔种植少量的肿瘤细胞，这个方法很适合观察微小转移灶。

在本研究中，我们没有选取单克隆而是选择了多克隆CPEΔN高表达细胞株。作者认为这种方式可以更直接的体现CPEΔN蛋白过表达赋予细胞的功能改变，而不是某一株单克隆细胞系的个性化行为，并且这种筛选方式可以节约成本，节省时间^[22, 23]^。分析荧光素酶报告基因检测结果，可以看到H1299-CPEΔN和H1299-control两个细胞株降解荧光素酶底物的能力没有差异。这个工作的完成将为进一步确证CPEΔN促进肺癌转移提供动物水平佐证。CPEΔN蛋白有希望成为新的肺癌转移预测分子，用于肿瘤的个性化诊断和治疗。

## References

[1] Skalka N, Caspi M, Caspi E (2013). Carboxypeptidase E: a negative regulator of the canonical Wnt signaling pathway. Oncogene.

[2] Cawley NX, Wetsel WC (2012). New roles of carboxypeptidase E in endocrine and neural function and cancer. Endocr Rev.

[3] Bachtiary B, Boutros PC, Pintilie M (2006). Gene expression profiling in cervical cancer: an exploration of intratumor heterogeneity. Clin Cancer Res.

[4] Cawley NX, Rodriguez YM, Maldonado A (2003). Trafficking of mutant carboxypeptidase E to secretory granules in a beta-cell line derived from Cpe(fat)/Cpe(fat) mice. Endocrinology.

[5] Chen H, Jawahar S, Qian Y (2001). Missense polymorphism in the human carboxypeptidase E gene alters enzymatic activity. Hum Mutat.

[6] Coleman DL, Eichler EM (1990). Fat (fat) and tubby (tub), two autosomal recessive mutations causing obesity syndromes in the mouse. J Hered.

[7] Cheng Y, Cawley NX, Loh YP (2014). Carboxypeptidase E (NF-α1): a new trophic factor in neuroprotection. Neurosci Bull.

[8] Hong Y, Ho KS, Eu KW (2007). A susceptibility gene set for early onset colorectal cancer that integrates diverse signaling pathways: implication for tumorigenesis. Clin Cancer Res.

[9] Cool DR, Normant E, Shen F (1997). Carboxypeptidase E is a regulated secretory pathway sorting receptor: genetic obliteration leads to endocrine disorders in Cpe(fat) mice. Cell.

[10] Fricker LD, Snyder SH (1983). Purification and characterization of enkephalin convertase, an enkephalin-synthesizing carboxypeptidase. J Biol Chem.

[11] Tang SS, Zhang JH, Liu HX (2009). PC2/CPE-mediated pro-protein processing in tumor cells and its differentiated cells or tissues. Mol Cell ndocrinol.

[12] He P, Varticovski L, Bowman ED (2004). Identificationof carboxypeptidase E and gamma-glutamyl hydrolase as biomarkers for pulmonary neuroendocrine tumors by cDNA microarray. Hum Pathol.

[13] Cutcliffe C, Kersey D, Huang CC (2005). Clear cell sarcoma of the kidney: up-regulation of neural markers with activation of the sonic hedgehog and Akt pathways. Clin Cancer Res.

[14] Walboomers JM, Jacobs MV (1999). Human papillomavirus is a necessary cause of invasive cervical cancer worldwide. J Pathol.

[15] Kim HJ, Hong J, Yoon HJ (2014). Carboxypeptidase E is a novel modulator of RANKL-induced osteoclast differentiation. Mol Cell.

[16] Murthy SR, Pacak K, Loh YP (2010). Carboxypeptidase E: Elevated expression correlated with tumor growth and metastasis in pheochromocytomas and other cancers. Cell Mol Neurobiol.

[17] Du J, Keegan BP, North WG (2001). Key peptide processing enzymes are expressed by breast cancer cells. Cancer Lett.

[18] Lee TK, Murthy SR, Cawley NX (2011). An N-terminal truncated carboxypeptidase E splice isoform induces tumor growth and is a biomarker for predicting future metastasis in human cancers. J Clin Invest.

[19] Mitka M (2011). Researchers discover new biomarker that may improve cancer care strategies. JAMA.

[20] Zhou K, Liang HY, Liu Y (2013). Overexpression of CPE-ΔN predicts poor prognosis in colorectal cancer patients. Tumor Biol.

[21] Liang XH, Li LL, Wu GG (2013). Upregulation of CPE promotes cell proliferation and tumorigenicity in colorectal cancer. BMC Cancer.

[22] Yang SY, Xiao JJ, Zhao DM (2014). Construction of lentivirus expression vector of miRNA-491-5p and screening of cell lines for stable expression of miRNA-491-5p. Nantong Da Xue Xue Bao (Yi Xue Ban).

